# Reproducibility of quantitative RT-PCR array in miRNA expression profiling and comparison with microarray analysis

**DOI:** 10.1186/1471-2164-10-407

**Published:** 2009-08-28

**Authors:** Yongxin Chen, Jonathan AL Gelfond, Linda M McManus, Paula K Shireman

**Affiliations:** 1 Department of Surgery, University of Texas Health Science Center, San Antonio, TX 78229, USA; 2 Department of Surgery, South Texas Veterans Health Care System, San Antonio, TX 78229, USA; 3 Department of Epidemiology and Biostatistics, University of Texas Health Science Center, San Antonio, TX 78229, USA; 4 Department of Pathology, University of Texas Health Science Center, San Antonio, TX 78229, USA; 5 Department of Periodontics, University of Texas Health Science Center, San Antonio, TX 78229, USA; 6 Department of Medicine, University of Texas Health Science Center, San Antonio, TX 78229, USA; 7 Sam and Ann Barshop Institute for Longevity and Aging Studies, University of Texas Health Science Center, San Antonio, TX 78229, USA

## Abstract

**Background:**

MicroRNAs (miRNAs) have critical functions in various biological processes. MiRNA profiling is an important tool for the identification of differentially expressed miRNAs in normal cellular and disease processes. A technical challenge remains for high-throughput miRNA expression analysis as the number of miRNAs continues to increase with *in silico *prediction and experimental verification. Our study critically evaluated the performance of a novel miRNA expression profiling approach, quantitative RT-PCR array (qPCR-array), compared to miRNA detection with oligonucleotide microchip (microarray).

**Results:**

High reproducibility with qPCR-array was demonstrated by comparing replicate results from the same RNA sample. Pre-amplification of the miRNA cDNA improved sensitivity of the qPCR-array and increased the number of detectable miRNAs. Furthermore, the relative expression levels of miRNAs were maintained after pre-amplification. When the performance of qPCR-array and microarrays were compared using different aliquots of the same RNA, a low correlation between the two methods (r = -0.443) indicated considerable variability between the two assay platforms. Higher variation between replicates was observed in miRNAs with low expression in both assays. Finally, a higher false positive rate of differential miRNA expression was observed using the microarray compared to the qPCR-array.

**Conclusion:**

Our studies demonstrated high reproducibility of TaqMan qPCR-array. Comparison between different reverse transcription reactions and qPCR-arrays performed on different days indicated that reverse transcription reactions did not introduce significant variation in the results. The use of cDNA pre-amplification increased the sensitivity of miRNA detection. Although there was variability associated with pre-amplification in low abundance miRNAs, the latter did not involve any systemic bias in the estimation of miRNA expression. Comparison between microarray and qPCR-array indicated superior sensitivity and specificity of qPCR-array.

## Background

MicroRNAs (miRNAs) are small noncoding RNAs of 20-22 nucleotides in length that direct posttranscriptional regulation through specific recognition of short sequences of target mRNAs, often in the 3' untranslated region, reviewed in [1-3]. With >200 members per species in higher eukaryotes, miRNAs are one of the largest gene families, accounting for ~1% of the genome [4]. Multiple studies have demonstrated that miRNAs are involved in numerous integral biological processes including development, cell proliferation, differentiation and apoptosis, reviewed in [5,6]. The complex and dynamic expression profiles of miRNA reflect the important roles in the control of mammalian growth and development. In fact, miRNA expression studies have demonstrated that aberrant expression of miRNAs is causally related to a variety of disease states such as cancer, diabetes, and heart failure [7-10]. As the number of miRNAs continues to increase with *in silico *prediction and experimental verification, miRNA profiling remains an important tool for identification of differential expression of miRNAs in normal and pathophysiologic processes.

Three main techniques have been used to detect and quantify miRNA including cloning, hybridization with selective probes, and polymerase chain reaction (PCR)-based detection, reviewed in [11]. Cloning strategies identified many of the initial miRNAs and revealed that miRNAs are present in many species [12-14]. Hybridization techniques include Northern blotting [15,16], bead-based flow-cytometry [17], and oligonucleotide microchip (microarray) [10,18,19]. While Northern Blotting using radioactive probes is very sensitive, disadvantages of Northern Blotting and cloning techniques are that both are time-consuming and impractical for large scale detection of hundreds of miRNAs. Microarrays have been widely used in miRNA profiling and can simultaneously determine expression levels for large numbers of miRNAs in a single experiment. However, the short length of miRNAs with inherently different melting temperatures (Tm) and the highly similar sequences between miRNA family members make probe design more difficult than for mRNA arrays [20,21]. Although the development of chemically modified probe design, such as Locked nucleic acid (LNA) and 2'-O-(2-methoxyethyl)-(MOE) can elevate Tm and stabilize hybridization [22,23], synthesis and chemical modification of RNA probes can be costly. While potentially providing more specific signals than microarray, bead-based flow cytometric miRNA profiling methods require the removal of genomic DNA, followed by recovery of small RNA fragments [17]; these multiple steps have the potential to alter the miRNAs present in the original sample. Furthermore, large quantities of RNA are needed for hybridization techniques, which limit their use in samples that yield small amounts of RNA. PCR-based techniques are able to detect low copy numbers of individual miRNAs with high sensitivity and specificity on both the precursor [24,25] and the mature form of miRNAs [26]. The stem-loop reverse transcription- polymerase chain reaction (RT-PCR) can profile miRNA expression with only nanograms of total RNA or even single cells [26-28]. Recently a quantitative RT-PCR based array method (qPCR-array) became available which combined the high sensitivity provided by the stem-loop RT-PCR with the ability to profile large numbers of miRNA in a single experiment [29].

The current study critically evaluated the reproducibility of qPCR-array analyses using multiple aliquots of the same RNA sample obtained from proliferating murine myoblast cells to determine the intra- and inter-assay reproducibility. The effect of pre-amplification on qPCR-array reproducibility and sensitivity was also evaluated. Finally, false positive rates of differential miRNA expression were compared to the microarray technique.

## Methods

### Cell culture and RNA isolation

Mouse C2C12 myoblasts [American Type Culture Collection (ATCC), Manassas, VA] were cultured in growth media containing Dulbecco's Modified Eagle Medium (DMEM) (ATCC), supplemented with 10% fetal bovine serum (FBS) (Hyclone, Logan, UT) and 1% penicillin-streptomycin (Mediatech, Inc., Herndon VA) at 37°C in a humidified atmosphere of 5% CO_2 _in air. Total RNA was isolated from proliferating C2C12 cells using TRIzol (Invitrogen, Carlsbad, CA) according to manufacturer's instructions. Total RNA concentration was quantified by absorbance at 260 nm using a SmartSpec 3000 spectrophotometer (Bio-Rad, Hercules, CA). RNA integrity was assessed using the Agilent 2100 Bioanalyzer (Agilent Technologies, Santa Clara, CA) and samples with RNA integrity number (RIN) >9 were used in the array studies. Aliquots of the same RNA sample were used for all the experiments in both the microarray and qPCR-array measurements.

### TaqMan Real-time PCR microRNA Array

The stem-loop RT-PCR based TaqMan Rodent MicroRNA Arrays (Applied Biosystems, Foster City, CA) were used representing 585 mature miRNA present in the Sanger miRBase v12 in a two-card set of arrays (Array A and B). Each array contains six positive controls and one negative control. Array A focused on more highly characterized miRNAs, while Array B contains many of the more recently discovered miRNAs along with the miR* sequences. RT-PCR reactions were performed according to the manufacturer's instructions. All reagents were obtained from Applied Biosystems. Briefly, 500 ng of total RNA obtained from proliferating C2C12 cells was reverse-transcribed using Megaplex RT Primers and the TaqMan miRNA reverse transcription kit in a total of 7.5 μl volume. Quantitative real-time PCR was performed using Applied Biosystems 7900HT system and a TaqMan Universal PCR Master Mix with 6 μl cDNA input per plate. Cycle threshold (Ct) values were calculated using the SDS software v.2.3 using automatic baseline settings and a threshold of 0.2. Since a Ct value of 35 represents single molecule template detection, Ct values > 35 were considered to be below the detection level of the assay [30]. Therefore, only the miRNAs with a Ct ≤ 35 were included in the analyses. PCR efficiency (E) was calculated according to the formula: *E *= 10^-1/slope^, as previously described [31]. The Ct value of an endogenous control gene (MammU6) was subtracted from the corresponding Ct value for the target gene resulting in the ΔCt value which was used for relative quantification of miRNA expression. As there is an inverse correlation between ΔCt and miRNA expression level, lower ΔCt values were associated with increased miRNA expression.

### Pre-amplification of miRNA cDNA

For pre-amplification, 150 ng of total C2C12 RNA was reverse-transcribed and the product (2.5 μl) was pre-amplified using Megaplex PreAmp Primers and TaqMan PreAmp Master Mix (Applied Biosystems) in a 25 μl PCR reaction. The pre-amplification cycling conditions were 95°C for 10 min, 55°C for 2 min and 75°C for 2 min followed by 12 cycles of 95°C for 15 sec and 60°C for 4 min. The pre-amplified cDNA was diluted with 0.1× TE (pH 8.0) to 100 μl and then 10 μl diluted cDNA was used in each plate for real-time PCR reactions.

### MiRNA microarray analysis

The miRNA microarray analysis was performed by LC Sciences (Houston, TX) using two replicate aliquots of the C2C12 RNA. Briefly, the assay started with 8 μg of total RNA. After size fractionation of the RNAs using a YM-100 Microcon centrifugal filter from Millipore, poly(A) tails were added to RNA sequences with lengths less than 300 nucleotides using poly(A) polymerase. An oligonucleotide tag was ligated to the poly(A) tail for later fluorescent dye staining. RNA samples were hybridized overnight on a μParaflo microfluidic chip using a micro-circulation pump developed by Atactic Technologies [32]. Each microfluidic chip contained the following probes: 1) detection probes which consisted of chemically-modified nucleotide sequences complementary to 617 mouse mature miRNAs listed in the Sanger miRBase Release 12.0; 2) a total of 49 positive and negative control probes designed by LC Sciences to determine uniformity of sample labeling and assay conditions and 3) a spacer segment of polyethylene glycol to extend the coding segment away from the substrate. The probes were made *in situ *using photogenerated reagent chemistry. The hybridization melting temperatures were balanced by chemical modifications of the probes. Hybridization reactions were performed in 100 μl 6×SSPE buffer (0.90 M Sodium Chloride, 60 mM Sodium Hydrogen Phosphate, 6 mM EDTA, pH 6.8) containing 25% formamide at 34°C. After RNA hybridization, tag-conjugating Cy3 dyes (one-color hybridization) were circulated to samples for dye staining. Each analyzed miRNA was repeated five times. A GenePix 4000B (Molecular Device, Union City, CA) laser scanner was used to collect the fluorescence images which were digitized using Array-Pro image analysis software (Media Cybernetics, Bethesda, MD).

### Data analysis

MiRNA microarray data were analyzed by LC Sciences by subtracting the background and normalizing the signals using a Locally-weighted Regression filter by 5S rRNA, as described previously [33]. A miRNA was listed as detectable when it met at least three criteria: 1) signal intensity higher than 3× the background standard deviation, 2) spot coefficient of variation (CV) < 0.5, in which CV was calculated as (standard deviation)/(signal intensity), and 3) at least 50% of the repeated probes had a signal 3-times higher than background standard deviation. The miRNA microarray data used the total gene signal, which was proportional to the total number of targets bound by the probes targeting each miRNA. Differentially expressed signals were determine by t-test with *p *< 0.01. To compare qPCR-array and microarray assays, the log2 of microarray signals was used.

Differential expression of miRNA measured by the qPCR-array was tested using the Welch t-test. A *p *value < 0.01 was considered significant. Fisher's Z transformation was used to test the equivalence of correlations [34]. The power calculation was performed assuming unequal variances with estimates of the standard deviation based upon all replicates in the experiment; estimates for the standard deviations were on the log_2 _scale and included miRNAs that had Ct values of ≤35. The PROC POWER function in SAS 9.1 (Cary, NC) was used for this calculation. The Type I (false positive error) error rate was chosen conservatively as <0.0005 so that a Bonferroni adjustment for 100 hypotheses would achieve a family-wise error rate of 0.05.

Both miRNA microarray and qPCR-array data were submitted to the Gene Expression Omnibus (GEO; ) in minimum information about a microarray experiment (MIAME) compliant format with the series accession number GSE16000.

## Results

### Reproducibility of qPCR-array

**Figure 1:**
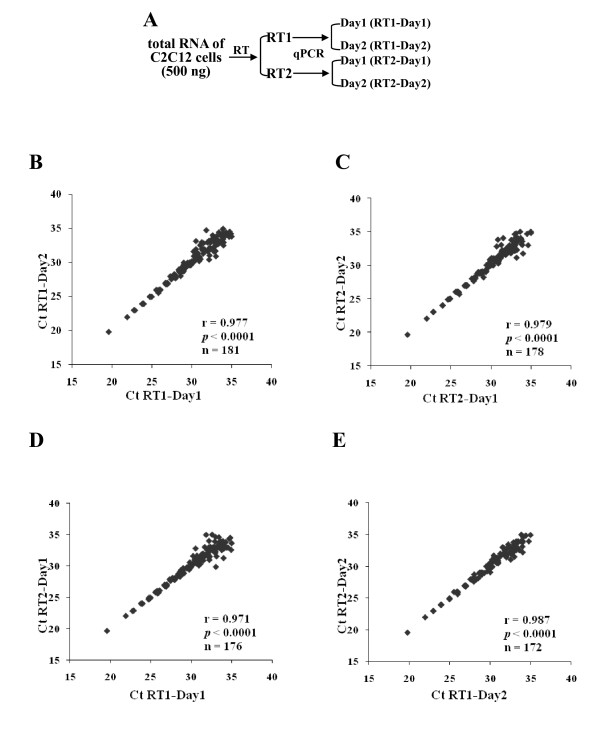
**Experimental design and reproducibility of qPCR-array**. A, The same RNA (500 ng) was used in two different reverse transcription (RT) reactions and the products were used in qPCR-arrays performed on different days. B and C, inter-assay reproducibility as determined by comparison of raw Ct values between replicates of different day reactions. D and E, inter-sample reproducibility as determined by comparison of raw Ct values between replicates of different RT reactions. Corresponding r and *p *values were determined by linear regression analysis. Sample number presented in each plot.

Many *in vitro *handling steps are required for array analyses and each processing step has the potential to generate variability in the data. Replicate preparations using different aliquots of a single C2C12 RNA sample (500 ng) were used to determine the reproducibility of the reverse transcription process as well as the results of the same reverse transcription products performed on different days. TaqMan Array A, which quantifies 375 of the most highly characterized rodent miRNAs, was used. To evaluate the reproducibility of reverse transcription and PCR reactions, different aliquots of the same RNA were used for two separate reverse transcription reactions, and the products of each reaction were used in two separate assays on two separate days for a total of 4 assays (Figure 1A). 47% of the miRNAs had average Ct values ≤35 (see Additional file 1). The raw Ct values of detectable miRNAs (miRNAs with Ct ≤35) were compared between each replicate for a total of 4 conditions. The comparison for every two replicates demonstrated strong correlation with an average correlation coefficient 0.978 (Figure 1B-E). The correlation for each replicate set was significant (*p *< 0.0001) which indicated a high degree of reproducibility. The slope of the linear trendline fitted along the correlation plot was nearly equal to 1 in each comparison. The correlation coefficient between replicates from the same or different reverse transcription reactions were similar suggesting that different reverse transcription reactions did not introduce significant variation. Similar results were obtained when ΔCt values were used for the comparison (data not shown). PCR amplification efficiency was determined using a series of different C2C12 RNA concentrations (5000 ng, 500 ng, or 50 ng). PCR efficiency for miRNAs with Ct ≤ 35 (n = 87) ranged between 2.22 and 1.80 with an average efficiency of 1.94 ± 0.09.

An important consideration in experimental design is determining the number of samples needed to provide statistically valid results. Power calculations using the above data indicated that at least four replicates are needed to obtain >90% power with a Type I error rate of <0.0005 to detect a fold change of 2 or 4 given the observed standard deviations (Std Dev) (Table 1).

**Table 1:** Power Analysis of qPCR-array data derived from replicates of the same C2C12 RNA sample.

Fold Change	Std Dev	% miRNA*	Type I Error Rate	Actual Power	Number Per Group
2	0.1	17%	0.00025	0.944	4
2	0.2	58%	0.00034	0.974	6
2	0.3	82%	0.00039	0.937	8
4	0.1	17%	0.00025	>.999	4
4	0.2	58%	0.00025	0.944	4
4	0.3	82%	0.00030	0.974	5

*Percent of miRNA in our sample with the stated power.

### Reproducibility and Reliability of qPCR-array with pre-amplification

**Figure 2:**
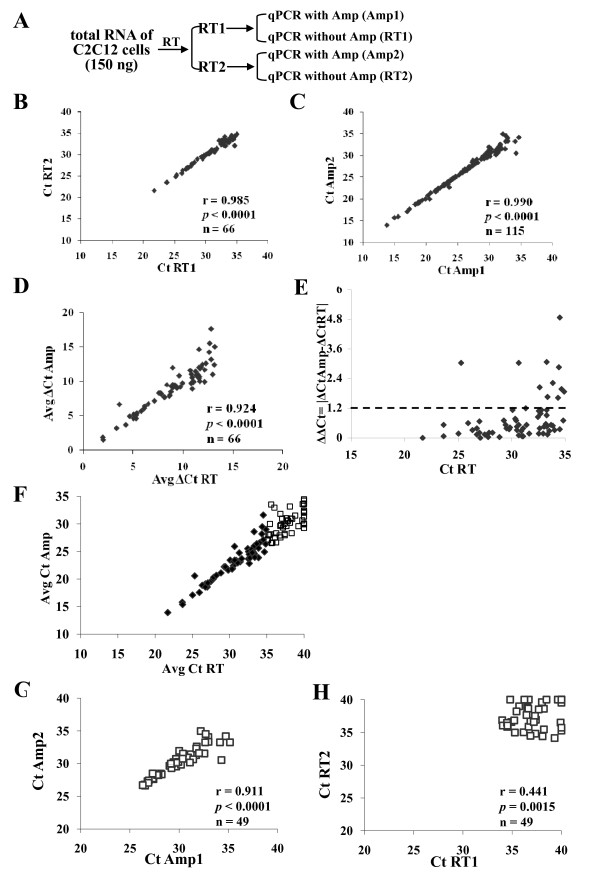
**Experimental design and comparison of miRNA expression between samples with and without pre-amplification**. A, The same RNA (150 ng) was used in the reverse transcription (RT) and pre-amplification reactions. B and C, correlation plot between raw Ct values for each replicate with (Amp) or without (RT) pre-amplification. D, correlation plot of ΔCt obtained with and without pre-amplification. E, difference in miRNA expression (ΔΔCt) between amplified and non-amplified samples. ΔΔCt values were plotted as the average Ct of non-amplified samples. F, correlation plot between raw Ct values obtained with and without pre-amplification. Data points represented by white dots indicate miRNAs that were only detectable in pre-amplified samples. G and H, correlation plot of Ct values of miRNAs detected only in pre-amplified samples between replicates with and without pre-amplification. Corresponding r and *p *value determined by linear regression analysis and sample number presented in each plot.

Pre-amplification could potentially bias the expression of miRNA transcripts. Therefore, it was important to determine if the relative expression of miRNAs in the original cell population was uniformly maintained after pre-amplification. To assess the reliability of pre-amplification, miRNA expression profiles obtained with and without pre-amplification using MiRNA TaqMan Array B, which contains 210 recently discovered miRNAs along with minor miR* sequences, were compared [35]. Different aliquots of the same C2C12 RNA (150 ng) were used in the reverse transcription and pre-amplification reactions, both in duplicate (Figure 2A). The raw Ct values of detectable miRNAs measured in each replicate qPCR-array were highly correlated (Figure 2B and 2C). The Pearson's correlation coefficients for each replicate qPCR-array pair comparison was 0.985 and 0.990 for qPCR-arrays without and with pre-amplification, respectively. To evaluate if pre-amplification introduced bias to the original miRNA expression levels, ΔCt values were compared between amplified and non-amplified miRNAs and displayed on the correlation plot in Figure 2D. Only miRNAs with a Ct ≤ 35 in samples without pre-amplification were included and the calculated correlation was 0.924. A paired t-test was used to test the null hypothesis of zero-difference in the means and demonstrated that there was no observed systematic bias in the estimation of the miRNA expression levels (t = -0.63, *p *= 0.26). Therefore, miRNA quantification using pre-amplification resulted in relative miRNA expression levels that represented the level in the cell population without pre-amplification.

Specific miRNA pre-amplification uniformity was measured by calculating the ΔCt from all miRNAs with Ct ≤ 35 and determining the ΔΔCt between amplified (Amp) and non-amplified reverse transcription (RT) targets (ΔΔCt = |ΔCt_Amp_-ΔCt_RT_|). ΔΔCt values near zero therefore indicate pre-amplification uniformity. Targets that produce ΔΔCt values within ± 1.5 were considered uniformly preamplified (TaqMan PreAmp Master Mix Protocol, Applied Biosystems). Using this approach, 85% miRNAs displayed ΔΔCt < 1.2 (Figure 2E). When the Ct was ≤ 30, 95% of all miRNAs had a ΔΔCt < 1.2.

To assess the efficiency of pre-amplification, the number of detectable miRNAs with Ct ≤ 35 in each replicate of both qPCR-arrays with and without pre-amplification was estimated. The average Ct values of detectable miRNAs in amplified samples were plotted against those obtained from samples that were not pre-amplified (Figure 2F). The number of detectable miRNAs was almost two-fold higher in pre-amplified samples (115/216, 53%) compared to samples without pre-amplification (66/216, 31%). All miRNAs detected in non-amplified samples were also detected in samples with pre-amplification. This difference demonstrates the increased sensitivity of pre-amplification prior to qPCR-array analysis. The Ct values of the miRNAs only detected by pre-amplification showed higher reproducibility for the pre-amplified samples compared to samples that were not pre-amplified (r = 0.991 vs. r = 0.441; *p *< 0.0001) (Figure 2G and 2H). This demonstrates that pre-amplification improved not only the number of miRNAs that were detectable (Ct ≤ 35), but pre-amplification also increased the reliability of these miRNA measurements. Since PCR products double with each amplification cycle, a 12-cycle pre-amplification will result in 12 cycles of Ct shift, or a 2^12 ^= 4096-fold change. Because the cDNA was diluted by 24 times in the pre-amplified samples compared to samples without pre-amplification, theoretically, this allowed a 4096/24 = 171-fold difference of input between amplified and non-amplified samples or a decrement of 7.4 cycles of Ct. There were 7.6 ± 1.5 (mean ± Std Dev, n = 115) cycles decrement with the Ct value of the detectable miRNAs in samples with pre-amplification, indicating almost 100% pre-amplification efficiency.

### Comparison of qPCR-array and microarray assays

**Figure 3:**
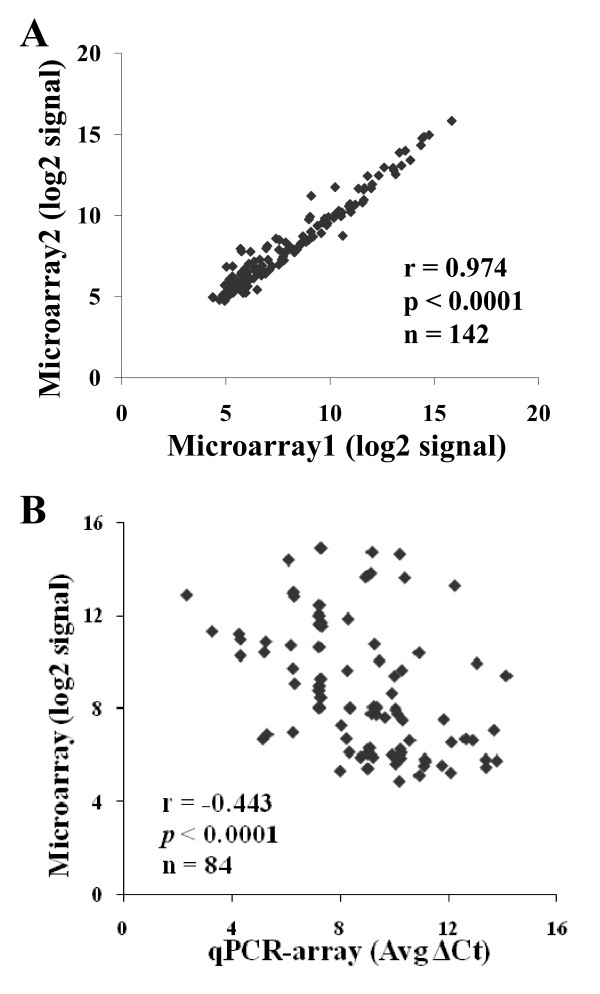
**Reproducibility of microarray and comparison of miRNA expression between microarray and qPCR-array**. A, correlation plot of log2 signals between the duplicate microarray assays. B, correlation plot comparing the ΔCt values of qPCR-array with the log2 of the microarray signal for each miRNA (n = 84). Corresponding r and *p *value determined by linear regression analysis.

Microarray is one of the most popular technologies for miRNA expression profiling [10,18,19]. The relationship between results obtained with qPCR-array and microarrays was determined. Independent miRNA expression profiling studies using uParaflo microfluidic biochips were performed by an independent company [36]. Aliquots of the same C2C12 RNA were used in both the qPCR-array (500 ng) and microarrays (8 μg). Comparison of miRNA expression level in duplicate samples for microarray analysis indicated good reproducibility (r = 0.974; Figure 3A). Since pre-amplification was not used in microarray assays, miRNA expression levels in microarray were compared to those in miRNA TaqMan Array A without pre-amplification; 84 miRNAs were detected in both microarray and qPCR-array (Ct ≤ 35). The results of both assays were compared by plotting the ΔCt values of qPCR-array versus the log2 of the microarray signal for each miRNA (Figure 3B). An inverse correlation should exist between the two methods since increasing miRNA expression was associated with decreasing ΔCt values in qPCR-array and increasing log2 signal in the microarray assay. The calculated correlation was -0.443. Although significant (*p *< 0.0001), this correlation indicated considerable variation in the results associated with the separate analysis platforms. To determine which miRNAs displayed the highest degree of variation for the two platforms, GC content and miRNA expression level were evaluated for each mature miRNA (Table 2). There were no significant differences in correlation coefficients when the detected miRNAs were divided into three groups based on GC content. However, individual correlation coefficients for subsets based on Ct value indicated a much higher variation between the two platforms for the low abundant miRNAs (Ct > 30) compared to the moderately (Ct between 25-30) and highly (Ct ≤ 25) expressed miRNAs.

**Table 2:** Comparison of miRNA expression between microarray and qPCR-array basedon GC content and Ct value

		Number of miRNA	Correlation coefficient	*p *value*
GC contents (%)	>30 to 45	25	-0.413	
	>45 to 55	41	-0.403	
	>55	18	-0.490	0.8988

Ct value	>20 to 25	9	-0.709	
	>25 to 30	55	-0.428	
	>30 to 35	20	-0.108	0.0428^†^

*Test of equivalence of correlations.

^†^*p *value for >25 to 30 compared to >30 to 35, >20 to 25 excluded from test due to small sample size in group.

To further compare the performance of qPCR-array and microarray, the expression levels for each miRNA in each replicate of the two platforms were compared. False positivity was defined as detection of significant differential expression of miRNAs between replicates in qPCR-array and microarrays. In-depth microarray data analyses were performed by the company and revealed 48 out of 382 miRNAs (13%) with significant differential expression (*p *< 0.01) between the aliquots of the same C2C12 RNA. However, only 2 (miR-503, 322) out of 172 detectable miRNAs (1%) were demonstrated to be significantly different (*p *< 0.01) between different reverse transcription reactions in qPCR-array. These two miRNAs were not detectable in the microarray assays (signal < 32). The 48 differentially expressed miRNAs in the microarray had a wide range of expression levels, including 23 miRNAs with signals >500 and 25 miRNAs with signal <500 (ranging between 10 and 15000 with average 1601 ± 2767). These results indicated that a low expression level was not always responsible for the false positive differential expression of miRNAs in the microarray analysis.

## Discussion

Due to the superior sensitivity and specificity, qPCR has been used as the "gold standard" to verify microarray expression data. The availability of qPCR-arrays makes this approach the method of choice for high-throughput miRNA expression profiling. Consistent with the results of Mestdagh *et al*. in profiling human miRNAs [29], our results clearly demonstrated high reproducibility of murine qPCR-array using RNA derived from C2C12 cells. Although Mestdagh et al [29] suggested reverse transcription reactions as a major factor contributing to the observed variation, our comparisons between different reverse transcription reactions with qPCR-arrays performed on different days produced similar correlation coefficients.

Pre-amplification has been used extensively for array analysis, especially when the RNA quantity is limited. By comparing miRNAs identified in samples with and without pre-amplification, our results demonstrated that the number of detectable miRNAs was almost twofold higher in pre-amplified samples. This result was very reproducible even when large amounts of RNA (500 ng) were used (Chen and Shireman, unpublished data). Thus, amplification improved the sensitivity of the qPCR-array analysis, enabling the identification of biologically regulated miRNAs that may have been below the detection level of the qPCR-array if pre-amplification had not been used. Similar results have also been demonstrated with PCR amplification using mRNA templates [37,38]. A major concern with using pre-amplification was the possibility that not all miRNA transcripts would be uniformly amplified. Our results suggested that relative miRNA expression levels were maintained between samples with and without pre-amplification. However, while most of the miRNAs (85%) were uniformly pre-amplified, we did observe increased variation associated with pre-amplification in low abundant miRNAs. Although primer and probe length/sequence could influence the pre-amplification efficiency, miRNAs with low expression level and high Ct values may also contribute to the variation. Similar uniformity of pre-amplification of miRNA has previously been reported by Mestdagh et al [29] except the correlation coefficient was decreased (r^2 ^= 0.797) for miRNA expression from amplified and non-amplified samples compared to the current study (r^2 ^= 0.854). Differing amounts of initial RNA used for pre-amplification, 10 ng (Mestdagh et al) versus 150 ng (current study) may account for the increased correlation coefficient in the current study. Therefore, the expression data for low abundant miRNAs obtained by pre-amplification should be interpreted with caution and confirmed in independent experiments or biological replicates.

To facilitate the comparison of miRNA expression data between different publications, it was important to determine the correlation between qPCR-arrays and microarrays. To our knowledge, this study is the first report with extensive comparison of miRNA expression between microarray and qPCR-array analysis. Even though the same RNA samples were used in both microarray and qPCR-array assays, a low correlation (r = -0.443) was observed with miRNA expression indicating the variation associated with the two platforms. Higher variation was especially observed in low abundant miRNAs. Stem-loop RT-PCR is highly sensitive and can detect an expression range of at least 7 logs [26], while microarray platforms can usually detect a 3-4 log of dynamic range [39,40]. Because the larger dynamic range imparts TaqMan qPCR-array with superior detection sensitivity, the expression variation observed in low abundant miRNA may reflect the different sensitivities of the two platforms. Although Ach et al [41] reported good correlations (r > 0.9) in 53/60 miRNAs when comparing microarray (Agilent) and individual TaqMan qPCR assays (Applied Biosystems), they could not compare all 60 miRNAs in one plot because qPCR and microarray assays have differential sensitivities to different miRNAs. When comparing the expression levels for each miRNA in each replicate of microarray and qPCR-array, we also observed a higher false positive rate of differential miRNA expression in the microarray assay. The stem-loop primers allow discrimination between miRNAs that differ by only a single nucleotide [26]. While the development of chemically modified probe design, such as Locked nucleic acid (LNA) and 2'-O-(2-methoxyethyl)-(MOE) enables high-affinity hybridizations to yield accurate miRNA detection [22,23], the lower false positive rate observed in qPCR-array may reflect its superior specificity compared to uParaflo microfluidic biochips. Although several papers reported high-resolution examination of the performance of microarrays in detecting differential expression at different fold changes [29,41], the results should be interpreted with caution and verified by quantitative RT-PCR due to the high rate of false positive results.

## Conclusion

Our studies demonstrated high reproducibility of TaqMan qPCR-array. Comparison between different reverse transcription reactions and qPCR-arrays performed on different days indicated that reverse transcription reactions did not introduce significant variation. The use of cDNA pre-amplification increased the sensitivity of miRNA detection. Although there were variations associated with pre-amplification in low abundance miRNAs, the latter did not involve any systemic bias in the estimation of miRNA expression ratios. Comparison between microarray and qPCR-array indicated superior sensitivity and specificity of qPCR-array.

## Authors' contributions

YC performed cell culture, RNA preparations and TaqMan qPCR-arrays. JG and YC performed the statistical analyses and organized the results. PKS and YC conceived this study, designed experiments, and revised the manuscript. LMM and PKS participated in the experimental design and the coordination of the work. All authors read, corrected and approved the final manuscript.

## Supplementary Material

Additional file 1 **Detectable miRNA list classified by Ct value**. The table provides the list of detectable miRNAs based on average Ct values. Click here for file
