# Supplementary material for: Reproducibility of quantitative RT-PCR array in miRNA expression profiling and comparison with microarray analysis
Source: BMC Genomics. 2009 Aug 28;10:407. doi: 10.1186/1471-2164-10-407 (PMC2753550; doi:10.1186/1471-2164-10-407)
Supplement: Additional file 1 — Detectable miRNA list classified by Ct value. The table provides the list of detectable miRNAs based on average Ct values. [file 1471-2164-10-407-S1.doc]

| **Additional file 1. miRNA list classified by Ct value** | |  |  |  |  |  |
| --- | --- | --- | --- | --- | --- | --- |
| **Ct** | **miRNAs** | | | | | |
| **≤ 20** | **Mammu6** | | | | | |
| **>20 to 25** | **16, 17, 19b, 20a, 24, 29a, 31, 106a, 222, 484** | | | | | |
| **>25 to 30** | **let7a, let7b, let7c, let7d, let7e, let7g, let7i, 9, 15a, 15b, 18a, 19a, 21, 23a, 26a, 26b, 27a, 27b, 28, 29c, 30a, 30b, 30c, 30d, 30e, 34b, 92a, 93, 99a, 99b, 100, 103, 106b, 125a, 125b, 130a, 132, 133b, 140, 143, 145, 146b, 151-3p, 152, 155, 182, 183, 186, 191, 193b, 195, 196b, 199a-3p, 210, 214, 221, 296-5p, 301a, 301b, 3230, 322, 328, 331, 335-3p, 342, 351, 365, 425, 486, 501-3p, 532-3p, 532-5p, 574-3p, 615-3p, 687** | | | | | |
| **>30 to 35** | **7a, let7f, 10a, 20b, 23b, 25, 29b, 34a, 34c, 98, 101a, 125a-3p, 125b-3p, 126-3p, 128a, 130b, 133a, 135b, 138, 139-5p, 142-3p, 146a, 148b, 150, 181a, 181c, 185, 187, 188-5p, 192, 193, 194, 197, 199a-5p, 202-3p, 218, 223, 224, 298, 324,-3p, -324-5p, 331-5p, 335-5p, 339-3p, 339-5p, 345-5p, 350, 361, 362-3p, 375, 376b, 379, 383, 409-3p, 423-5p, 449a, 449b, 450b-5p, 465a-3p, 465b-5p, 467a, 467b, 467c, 467d, 467e, 491, 494, 497, 500, 503, 509-3p, 542-5p, 582-5p, 590-5p, 652, 667, 669a, 671-3p, 674, 675-3p, 680, 682, 685, 708, 741, 743a, 744, 872, 881** | | | | | |
